# The Efficacy of Integrating Spirituality into Prenatal Care on Pregnant Women's Sleep: A Randomized Controlled Trial

**DOI:** 10.1155/2022/4295761

**Published:** 2022-02-03

**Authors:** Nahid Moradi, Azam Maleki, Saeedeh Zenoozian

**Affiliations:** ^1^School of Nursing and Midwifery, Zanjan University of Medical Sciences, Zanjan, Iran; ^2^Social Determinants of Health Research Center, Zanjan University of Medical Sciences, Zanjan, Iran; ^3^Department of Mental Health, Zanjan University of Medical Sciences, Zanjan, Iran

## Abstract

**Objective:**

The study is aimed at determining the efficacy of spiritual content counselling on improving the sleep quality and insomnia severity of pregnant women.

**Methods:**

This randomized controlled trial was carried out on 40 pregnant women recruited at five health centres of Abhar, Iran, 2020. The eligible women were allocated into two intervention and control groups according to the randomized blocking method. Group counselling with spiritual content was carried out in eight sessions at 16 to 20 weeks of gestation. The control group only received routine care. Data were collected using the Pittsburgh Sleep Quality Index (PSQI) and the Insomnia Severity Index (ISI) questionnaires in three stages, before the intervention, at 28, and 36 weeks of gestation. Statistical analysis was performed using repeated measure ANOVA test, chi-square, and independent *t*-tests. *P* < 0.05 was considered significant statistical level.

**Results:**

In the counselling group, the mean (SD) of a total score of sleep quality before the intervention was 9.45 (2.30) which decreased to 5.40 (1.56) in 36 weeks of gestational age, while in the control group was increased from 9.26 (2.15) to 11.47 (1.54). After the intervention based on the repeated measure ANOVA test, the mean total score of the insomnia severity, sleep quality, and its components decreased statistically in the second and third trimesters compared to the first trimester in the intervention group compared to the control group (*P* = 0.001).

**Conclusion:**

The results showed that counselling with spiritual content could effectively ameliorate sleep quality and reduce insomnia severity in pregnant women. It seems that the approach is an acceptable basis to design intervention programs in this field that can be considered by midwives. *Clinical Trial Registry and Registration Number*. The study was registered at the Iranian Registry of Clinical Trials under the IRCT20150731023423N15.

## 1. Background

One of the most common complaints during pregnancy is to have sleep disorders, such as insomnia, frequently waking up at night, high drowsiness during the day, mood swings, and unusual feelings during sleep [1]. In one review, more than 80% of pregnant women had sleep disorders [2]. A person's ability to fall asleep and the positive feeling after waking up indicate an ideal sleep quality [3]. The sleep quality varies in the different trimesters of pregnancy. Sleep disorders during pregnancy can increase unfortunate consequences, such as preeclampsia, preterm labor, miscarriage, urgent cesarean delivery, gestational diabetes, and postpartum depression [4–6].

To date, some studies have been conducted to improve sleep quality in pregnant women using a variety of pharmacological and nonpharmacological methods, each of which had different efficacy. The review of fourteen RCTs with 910 participants in Yan-Hua Lin's study shows that traditional Chinese medicines have significant effects on improving the Pittsburgh Sleep Quality Index [7]. In another study, Afrasiabian et al. show that the use of lemon verbena, an hour before bedtime for 4 weeks, could be improved significantly by the Pittsburgh Sleep Quality Index (PSQI) and Insomnia Severity Index (ISI) compared with the control group [8].

Today, religious beliefs are considered by many researchers as a treatment of mental and sleep disorders [9, 10]. According to the religious beliefs in Persian medicine, listening to music has a positive effect on sleep quality by impressing the soul, particularly when it is played within special position [11].

If spiritual health is at risk, loneliness, anxiety, and loss of meaning in life will be likely developed. However, strengthening the spirituality in patients allows them to adapt effectively to their problems. Therefore, being connected with higher power and support from spiritual or religious sources can improve one's quality of life, control mental health disorders, and create interpersonal support [10, 12]. In some studies, the use of spiritual counselling has been emphasized in the treatment of various aspects of pregnancy and childbirth, such as the stress during pregnancy and childbirth self-efficacy [13, 14]. Spirituality is a key factor in promoting maternal and fetal health [13–15]; it plays an important role in the acceptance of pregnancy [16]. According to studies, spiritual counselling has been effective in improving sleep quality for coronary artery disease patients [9] and the elderly [10]. Nevertheless, there is an information gap in the efficacy of spiritual content counselling in improving sleep quality for women during pregnancy. Given the importance of culture and religion in many countries and the role of midwives in identifying the attitudes and spiritual beliefs of the individuals, the study is aimed at determining the efficacy of spiritual content counselling on improving the sleep quality and insomnia severity of pregnant women.

## 2. Methods

### 2.1. Setting and Participants

The present study was a randomized control trial to determine the efficacy of counselling with spiritual content on improving prenatal sleep quality and sleep intensity in 2020. The study setting included 5 community health centres in Abhar, and the research population included pregnant women referred to the centres. Abhar is a city in Zanjan Province, in the northwest of Iran. Abhar has 5 health centres, and sampling had been done in the centres.

The sample size was calculated based on the sleep quality variable in Malikzadegan's study [17] using the formula for calculating the sample size in two independent groups. With considering 95% confidence interval (Z1‐*α* = 1.96), the test power of 80% (Z1‐*β* = 0.85), and the mean (standard deviation) of sleep quality in counselling group (*M*_1_ = 5.08, *S*_1_ = 4.24) and (*M*_2_ = 8.31, *S*_2_ = 1.86) in control groups, respectively, the total sample size was calculated to be 16 people. Due to the possibility of a 10% attrition in sample size, it increased to 20 in each group. Among 56 pregnant women evaluated by the first researcher, 40 women met the eligible criteria. The eligible pregnant women were assigned randomly to the two control and intervention groups based on the four-block design. In this way, six possible blocks will be assigned to group A (intervention) and group B (control). Then, the number of blocks was selected from the random number table until the study sample size reached. To ensure the concealment of the sequence of enrollment, we used the sequentially numbered, opaque sealed envelopes by a person who was not directly involved in the intervention.

**Figure 1:**
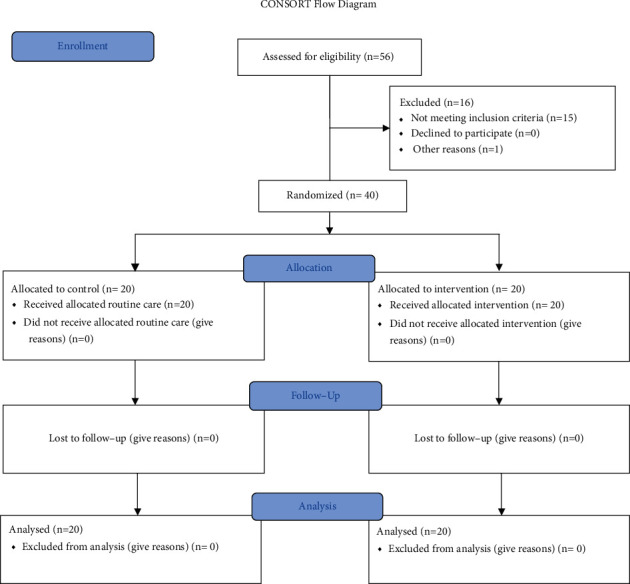
Chart of participant's enrollment and procedure.

The inclusion criteria were the age group of 18 to 35 years, the gestational age of 16-20 weeks, willingness to participate in the study, scoring more than 5 based on the Pittsburgh Sleep Quality Index (PSQI), and scoring less than 22 based on the General Health Questionnaire (GHQ-28). The exclusion criteria before randomization were consumption of sleeping pills, medical and obstetrics complications, and participation in a similar training program. There was no attrition in the study and after the interventions (Figure 1).

### 2.2. Procedure/Intervention

The spiritual content counselling was conducted in a place with a quiet and private setting for counselling in a health centre by the first author who is a midwife and familiar with group counselling under the supervision of a clinical psychologist. The content of counselling was developed by the research team based on the protocol of spiritual skills of Bolhari [18] and Asadi Zandi [19]. The number of the meeting was 8 sessions in two groups of 6 people and one group of 8 people. The meeting was held in 45–60 min for four following weeks (twice a week).

All the counselling sessions were attempted to use the principles and techniques of counselling to communicate effectively between the counsellor and participants. The counselling session was full of respect and intimacy and high self-confidence to provide an incredible opportunity for individuals to participate in group discussions. The content of each counselling session is listed in Table 1.

Data were completed by participants using the personal information questionnaire and the Pittsburgh Quality Sleep and the Insomnia Severity Index questionnaires in three stages, before the intervention, 28, and 36 weeks of gestational age.

### 2.3. Individual Information Questionnaire

The demographic questionnaire included age, level of education, employment status of the woman and her spouse, family income level, number of pregnancies, gestational age, number of abortions, pregnancy requirements, and residence status.

### 2.4. The Pittsburgh Sleep Quality Index (PSQI)

The Pittsburgh Quality Sleep Questionnaire was used to measure sleep quality over the past month with seven components: subjective sleep quality, sleep latency, sleep duration, habitual sleep efficiency, sleep disturbances, use of sleeping medication, and daytime dysfunction. Scoring the questions on a Likert scale ranges from zero to three. The total score ranges from zero to 21. People who score five or less are considered to have a good sleep quality. The reliability of the Persian version of the questionnaire was 0.65. Furthermore, the content validity was acceptable (CVI = 0.78 and CVR = 0.90) [20].

### 2.5. Insomnia Severity Index (ISI)

The Insomnia Severity Index is a brief self-report tool that assesses patient's perception of the intensity of insomnia. This index has seven questions [21]. Scoring questions on 5 Likert scales ranges from zero (never) to 4, so the score range is between 0 and 28. The total score of the questionnaire is obtained by adding the score of the items. Higher scores indicate the high intensity of insomnia. The reliability of Persian version in Chehri et al.'s had good reliability and validity (CVR = 0.77 and Cronbach's alpha 0.84) [21, 22].

### 2.6. The General Health Questionnaire (GHQ-28)

The 28-question form of the General Health Questionnaire (GHQ-28) was developed by Goldberg and Hiller in 1979. GHQ-28 scoring is from zero to 3. If the total score of the questionnaire is more than 23, it indicates a disorder in general health. Cronbach's alpha coefficient of the Persian version of the questionnaire in the study of Shayan et al. was reported to be 0.90 [23]. We used the GHQ-28 questionnaire to find eligible people, and this questionnaire was used only once in the registration phase.

### 2.7. Statistical Methods

Data were analyzed using SPSS software version 16. The normality of the data was examined using the Kolmogorov-Smirnov test; all variables had a normal distribution. To compare the demographic characteristics, we used the chi-square test, and to compare the sleep quality and the insomnia severity between the two groups, we used independent *t*-tests. The effect of group, time, and interaction between time and group was determined using the repeated measure ANOVA test. *P* < 0.05 was considered significant statistical level.

## 3. Result

### 3.1. Baseline Data

The mean age of women in the intervention group was 26.65 years, and in the control group was 28.55 years; the mean gestational age was 18.60 in the intervention group and 18.20 in the control group. Comparing the gestational age and age of women between the two groups was not statistically significant (*P* = 0.15). The results of Table 2 show that the comparison of individual profile variables in the two groups was not statistically significant, so the two groups were homogeneous (*P* = 0.31) (Table 2).

### 3.2. Sleep Quality

In the counselling group, the mean (SD) of a total score of sleep quality before the intervention was 9.45 (2.30) which decreased to 5.40 (1.56) in 36 weeks of gestational age. While the control group was increased from 9.26 (2.15) to 11.47 (1.54) at the same time. In the counselling group based on the repeated measure ANOVA test, the total mean score of sleep quality and its components significantly decreased after the intervention compared to the control group (*P* = 0.001). Furthermore, neither participant in the two groups used the sleep pills to control their sleep disorder (Table 3).

**Figure 2:**
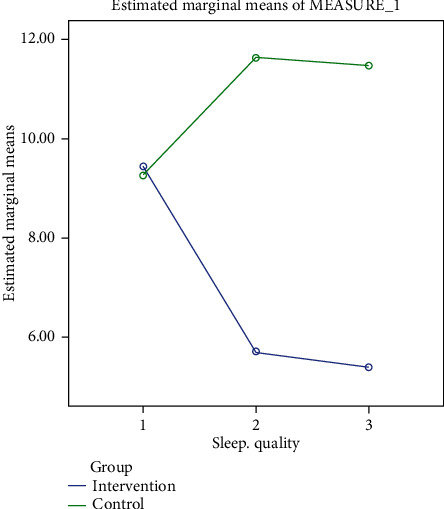
Chart of sleep quality in two groups.

A comparison of the mean scores of sleep quality between the two groups in three time periods is shown in Figure 2.

### 3.3. Insomnia Severity

In the counselling group, the mean total score of insomnia severity after the intervention was decreased in 36 weeks of gestational age compared to the control group (*P* = 0.001). Based on the repeated measure ANOVA test, the total mean score of insomnia severity in the second and third trimesters decreased compared to the control group. The observed differences between the two groups were statistically significant (*P* = 0.001) The effect size of intervention (“Eta” score) at the end of the follow-up period was about 0.66; it shows that counselling with a spiritual content was associated with a 66% reduction in the severity of insomnia (Table 4).

**Figure 3:**
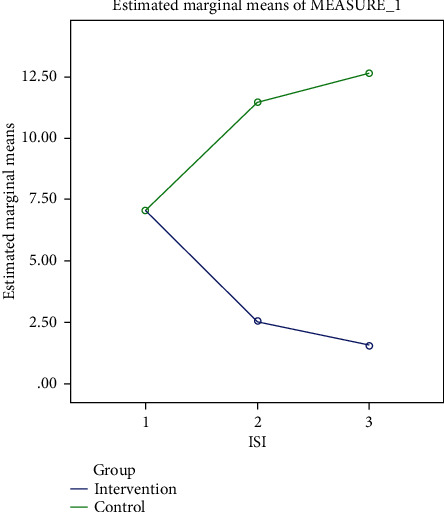
Chart of Insomnia Severity Index (ISI) in two groups.

A comparison of the mean scores of insomnia severity between the two groups in three time periods is shown in Figure 3.

## 4. Discussion

The study is aimed at determining the efficacy of spiritual content counselling on improving the sleep quality and insomnia severity of pregnant women. The results of the present study showed that counselling with spiritual content was effective in improving the sleep quality in pregnant women. The efficacy of the intervention continued to the second and third trimesters. Although no available studies were showing the effect of spiritual content counselling on sleep quality during pregnancy, the positive effects of spiritual counselling on improving sleep quality have been reported in the nonpregnant research community. Taheri et al. (2013) showed that after spiritual care based on the “Ghalb Salim” nursing model, the mean score of sleep quality in patients with coronary artery disease had a statistically significant difference in comparison with the control group [9]. Soheyli et al. (2013) reported a similar result [24]. In the present study, spiritual content counselling had a significant effect on all areas of sleep quality, except for sleep efficiency. However, in the above studies, an improvement was observed in all areas of sleep quality.

Some studies have emphasized various beliefs about the biological and psychological effects of spiritual-oriented counselling. Spirituality, as a powerful factor, can influence one's attitude, cognition, and behaviour, and as a mediator, can affect one's thinking and evaluation process [19, 25]. In the Qur'an, God describes sleep with the word “Noum” and says in the ninth verse of Surah Naba “We have made your sleep a source of peace, the mentally and physically rejuvenation, and to rebuild the worn-out limbs and relieve any fatigue or discomfort”. From the content of the tenth verse of Surah Anfal, it is clear that sleep causes the elimination of fear and the feeling of security. This shows that sleep and a sense of security complete each other [26].

The peace of mind caused by religious activities (e.g., listening to religious sounds) has been emphasized to reduce anxiety during pregnancy [27]. Peace of mind and healthy sleep are essential in exuding positive hormones from the brain, improving protein metabolism, increasing the function of the immune system, and reducing the stress reactions in the body [19]. According to our result, another positive effect of spirituality-based counselling is the improvement of sleep quality during pregnancy, from which the providers can benefit in the form of maternity care service packages.

In the present study, we used religious sounds during relaxation and meditation exercises, which had similar effects on the results of mindfulness and relaxation-based interventions [28]. Mindfulness strategies play a role in reducing stress and regulating emotional response. For instance, yoga leads to communication with higher and superior powers. The results of the present study show that spiritual counselling as well as the two interventions listed are useful in reducing the cognitive and physical arousal associated with insomnia in pregnancy.

In the present study, part of the intervention focused on sleep health education and identifying sleep stimuli. It seems that increasing spiritual attention, along with health advice and behaviour modification, can have a positive effect on sleep quality.

The results of the present study showed that after the intervention, the mean score of the areas of subjective sleep quality, sleep latency, sleep duration, sleep disturbances, use of sleeping medication, and daytime dysfunction in the intervention group decreased significantly. In contrast, the score of these areas in the control group had a significant increase. Comparing the scores of sleep efficiency in the two groups was not statistically significant. Also, none of the participants in either group used medication to control sleep disorders. In a clinical trial study, Felder et al. examined the efficacy of digital counselling using the cognitive-behavioural approach in treating the symptoms of insomnia among 208 pregnant women with a mean gestational age of 17 weeks. The intervention group received 6 sessions of online counselling per week in 5 areas (sleep restriction, stimulus control, cognitive therapy, relaxation techniques, and sleep hygiene training), and the control group received routine care. The follow-up period was 10 weeks after the end of the intervention. The results showed that the mean score of sleep quality and habitual sleep efficiency in the intervention group was significantly lower than the control group, while the mean score of sleep duration did not differ [29]. The results of the above study were consistent with the results of the present study in terms of overall sleep quality score, but not in terms of habitual sleep efficiency and sleep duration, which can be due to easy access to an online consultant.

The results of a review of 24 articles show that the mean score of sleep quality during pregnancy was 6.07. About 45.7% of pregnant women experience some degree of diminished sleep quality during pregnancy. However, changes in sleep quality score increase in the third trimester were 1.68 times higher than in the second trimester [2]. In the present study, the changes in the overall score of sleep quality in the control group were consistent with the results of the above study, so that the mean score of sleep quality of pregnant women increased from 9.26 in the first quarter to 11.47 in the third quarter. However, spiritual-oriented counselling reduced the overall score of sleep quality from 9.45 to 5.40. Therefore, using the present approach can be considered as a basis for improving sleep quality during pregnancy.

Religious activities can be a social determining factor in the state of sleep. So those who engage in religious activity are more likely to have a healthy sleep than those who do not. Based on the conceptual model proposed in a study, religious conflict may increase with hope, better support for mental health, a healthy lifestyle, reduced stress, healthier physiological function, limited substance use, and exposure to stress leading to healthy sleep [30]. However, conflicting results have also been reported. For example, Gillum (2013) showed that there was no significant relationship between participation in religious ceremonies in people over 40 years of age and sleep disorders [30], the results of which are inconsistent with the results of the present study because of the difference in the research population.

The results of the present study showed that spiritual content counselling was effective in improving the severity of insomnia in pregnant women. In the intervention group, the mean score of insomnia severity before the intervention decreased from 7.05 to 1.55 in the third trimester, and in the control group, it increased from 7.05 to 12.63. Comparing the mean scores in the three-time periods between the two groups was significant. So far, many interventions have been made to treat insomnia in pregnant women, but due to the adverse effects of drugs in pregnancy, researchers are trying to focus on nonpharmacological methods. The results of the present study are consistent with the results of some nonpharmacological interventions. As Jalal Marvi et al. showed (2019), distance education has positive effects on improving the intensity of insomnia in pregnant women [31]. In another study, Felder et al. (2020) showed that the implementation of 6 sessions of digital counselling based on the cognitive-behavioural approach could alleviate the intensity of insomnia in pregnant women [32]. Tomfohr-Madsen et al. reported similar results in 2017. In their study, holding 5 group counselling sessions based on the cognitive-behavioural approach had positive effects on alleviating the intensity of insomnia in pregnant women in the second trimester of pregnancy [33]. The findings of our study showed that integration of sleep hygiene training with spiritual orders can be as effective as other interventions.

It can be argued that some cognitive patterns, psychological characteristics, and behavioural patterns created by spiritual-oriented methods lead to improved health and improved physiological function of the body, followed by high psychological resistance in inappropriate physical and social situations. Accordingly, religious and spiritual practices lead to increased tolerance, patience, self-control, positivism, satisfaction, emotional control, optimism, self-efficacy (based on trust in God's blessing), altruism, kindness, and love [19]. The skill of “spiritual resilience” is another technique of spiritual counselling that encourages the audience to overcome physical and mental problems in a variety of ways.

### 4.1. Strengths of Study

All the principles of control trial studies were observed in this study, and we do not have a loss of following in participants. Data collection tools were standard, and psychometric properties of the Persian form of the questionnaires have been evaluated based on Iranian culture.

### 4.2. Limitations

Data collection was performed using a self-report questionnaire, and all participants were Muslim which should be considered in the generalizability of our findings. Also, the sample size was small, and the meeting was held without the attending of their spouse. Future studies with large sample sizes and is based on other cultures are required for a better conclusion.

### 4.3. Implications for Practice and/or Policy

In the present study, the effects of spiritual content counselling were not significant on habitual sleep efficiency. Given the importance of sleep in the physical and mental health of pregnant mothers, it is recommended that other psychological approaches be conducted in this field.

## 5. Conclusion

The results of the present study showed that counselling with spiritual content could effectively ameliorate sleep quality and reduce insomnia severity in pregnant mothers. Also, improving sleep quality continued until the third trimester of pregnancy. However, the control group had worse sleep quality with increasing gestational age than the intervention group. It seems that the approach of the present study is an acceptable basis to design intervention programs in this field that can be considered by researchers, consultants, and midwives.

## Figures and Tables

**Table 1:** Description of the content of spiritual counseling sessions.

Session 1	(i) Welcome
	(ii) Aims and objectives
	(iii) Pretest with Insomnia Severity Index (ISI) and the Pittsburgh Sleep Quality Index (PSQI)
	(iv) Defining of sleep, sleep quality, and preparing a list of stressful situations that affected sleep during pregnancy
	(v) Discussing the important of sleep during pregnancy
	(vi) Discussing the effect of stress on the body and sleep
	(vii) Educating sleep health program and life style modification for managing sleep problems during pregnancy
	(viii) Homework: writing the common pregnancy sleep problems and solutions

Session 2	(i) Encouraging to express their common pregnancy sleep problems and reviewing the solutions
	(ii) Discussing one's attitude towards spiritual issues, the role of God, and religion in her life
	(iii) Discussing one's experiences of reading the Qur'an and listening to the sound of the Qur'an in her calming and the soul
	(iv) Homework: writing the spiritual aspects of the pregnancy

Session 3	(i) Talking about the spiritual aspects of pregnancy, childbearing, and God's reward for pregnant women
	(ii) Reciting of verses and hadiths about sleep and the role of sleep comfortably in peace of mind
	(iii) Creating a spiritual environment with listening to the voice of the Quran in Surah Ar-Rahman (with the voice of Abdul Basit) for 5 min
	(iv) Discussing of one's feels after listening to the voice of the Quran
	(v) Homework: listening to the voice of the Quran and relax their muscles with deep breathing for getting rid of the stress/twice daily for 10 to 15 minutes

Session 4	(i) Reviewing the content of previous sessions and summarize members' opinions
	(ii) Blessings of God and the role of it in reinterpreting concept of pregnancy and overcoming the worrisome symptoms of pregnancy
	(iii) Discussing of prayer therapy to reduce the worries, get rid of some symptoms of pregnancy and to increase hope, resilience
	(iv) Homework: encouraging to create a daily spiritual space of time or place at home
	(v) Writing the experience of participating in religious programs or doing spiritual issues

Session 5	(i) Discussing one's experiences of participating in religious programs or doing spiritual issues
	(ii) Discussing on the role of charity, trust, recourse, patience, kindness, and forgiveness in managing emotions and life stressors
	(iii) Discussing on the role of gratitude to God in enduring hardships, increasing hope and self-confidence, adapting, and accepting the situation
	(iv) Encouraging to strengthen one's inner hope, locus of control, and resilience
	(v) Reciting the verses of Surah Maryam with the voices of the volunteer members with focusing its meaning
	(vi) Homework: writing down situations that make you feel guilty or angry about yourself or others

Session 6	(i) Discussing on one's experiences of forgiveness in life and anger towards the guilty
	(ii) Discussing the meaning of life with the birth of a baby
	(iii) Listening to “nature's music” the sound of birds, rivers and waterfalls with illustration hugging her baby
	(iv) Encouraging to participate in charity, kindness, and forgiveness
	(v) Homework: writing some strategies to cope with stress and improving sleep quality

Session 7	(i) Reviewing their strategies to cope with stress and improving sleep quality
	(ii) Designing a play of a stressful situation, charting a table of tensions, the severity of tension, behavior and attitudes, satisfaction with the coping method employed in those situations, and simple suggestions to cope with stress
	(iii) Homework: listening to the voice of the Quran and relax their muscles with deep breathing for getting rid of the stress/twice daily for 10 to 15 minutes

Session 8	(i) Reviewing the content of previous sessions and summarize members' opinions
	(ii) Discussing one's experiences of spiritual self-awareness, problem-solving skills with a spiritual approach

**Table 2:** Comparison of the frequency distribution of demographic characteristics between two study groups.

Social and midwifery characteristics	Intervention			Control	*P* value^∗^
	*N*	%	*N*	%	
Education					0.31
Guidance	3	15	7	35	
Diploma	13	65	9	45	
Academic	4	20	4	20	
Job					0.50
Unemployed	20	100	19	95	
Employed	0	0	1	5	
Family monthly income					0.42
Not enough	2	10	1	5	
To some degree enough	7	35	11	55	
Sufficient	11	55	8	40	
Housing status					0.74
Owner	8	40	7	35	
Rented	12	60	13	65	
Gravid					1
First pregnancy	10	50	10	50	
≥ two pregnancy	10	50	10	50	

^∗^Chi-square test.

**Table 3:** The comparison of sleep quality between the two groups in three stage (preintervention, 28, and 36 weeks of gestation).

Variables	Time	Intervention		Control		^∗∗^ *P* value	*F*	Eta	MD^∗∗∗^	^∗^ *P* value
		Mean	SD	Mean	SD					
Sleep quality Total score	Baseline	9.45	2.30	9.26	2.15	0.75	66.90	0.64	-3.93	0.001
	28^th^ w	5.70	1.55	11.63	2.06	0.001				
	36^th^ w	5.40	1.56	11.47	1.54	0.001				

Subjective sleep quality	Baseline	0.8	0.69	1.00	0.66	0.36	19.90	0.35	-0.96	0.001
	28^th^ w	0.3	0.47	1.58	0.69	0.001				
	36^th^ w	0.1	0.30	1.53	0.69	0.001				

Sleep latency	Baseline	1.75	0.63	1.57	0.60	0.39	37.49	0.50	-0.79	0.001
	28^th^ w	0.85	0.58	2.05	0.62	0.001				
	36^th^ w	0.80	0.41	2.15	0.60	0.001				

Sleep duration	Baseline	0.45	0.82	0.52	0.84	0.77	7.09	0.16	-0.51	0.002
	28^th^ w	0.1	0.30	0.73	0.87	0.001				
	36^th^ w	0	0	0.84	0.83	0.001				

Sleep efficiency	Baseline	3.0	0.01	2.94	0.22	0.31	1.05	0.02	0.08	0.31
	28^th^ w	3.0	0.01	2.89	0.45	0.31				
	36^th^ w	3.0	0.01	2.89	0.45	0.31				

Sleep disturbances	Baseline	0.90	0.30	0.89	0.31	0.95	6.07	0.14	-0.26	0.004
	28^th^ w	0.55	0.51	0.94	0.22	0.004				
	36^th^ w	0.60	0.50	1.0	0.01	0.001				

Daytime dysfunction	Baseline	0.7	0.57	0.78	0.71	0.66	24.46	0.41	-0.79	0.001
	28^th^ w	0.3	0.47	1.57	0.6	0.001				
	36^th^ w	0.2	0.41	1.21	0.53	0.001				

Sleeping medication	Baseline	0	0	0	0	0	—	—	—	—
	28^th^ w	0	0	0	0	0				
	36^th^ w	0	0	0	0	0				

^∗∗^Independent *t* test; ^∗^repeated measures ANOVA; ^∗∗∗^mean diff.

**Table 4:** The comparison of insomnia severity between the two groups in three stage (preintervention, 28, and 36 weeks of gestation).

Variables	Time	Intervention		Control		^∗∗^ *P* value	*F*	Eta	Mean diff	^∗^ *P* value
		Mean	SD	Mean	SD					
Insomnia severity	Baseline	7.05	4.29	7.05	4.19	0.99	8.79	0.66	-6.66	0.001
	28^th^ w	2.55	1.60	11.47	3.23	0.001				
	36^th^ w	1.55	0.94	12.63	3.7	0.001				

## Data Availability

The data sets used and analyzed during the current study are available from the corresponding author on reasonable request.

## Abbreviations

PSQI: The Pittsburgh Sleep Quality Index
ISI: The Insomnia Severity Index.
